# PSIONplus^m^ Server for Accurate Multi-Label Prediction of Ion Channels and Their Types

**DOI:** 10.3390/biom10060876

**Published:** 2020-06-07

**Authors:** Jianzhao Gao, Hong Wei, Alberto Cano, Lukasz Kurgan

**Affiliations:** 1 School of Mathematical Sciences and LPMC, Nankai University, Tianjin 300071, China; gaojz@nankai.edu.cn (J.G.); weihong96@mail.nankai.edu.cn (H.W.); 2 Department of Computer Science, Virginia Commonwealth University, Richmond, VA 23284, USA; acano@vcu.edu

**Keywords:** ion channel, ion channel type, voltage-gated ion channel, ligand-gated ion channel, sequential prediction, multi-label prediction

## Abstract

Computational prediction of ion channels facilitates the identification of putative ion channels from protein sequences. Several predictors of ion channels and their types were developed in the last quindecennial. While they offer reasonably accurate predictions, they also suffer a few shortcomings including lack of availability, parallel prediction mode, single-label prediction (inability to predict multiple channel subtypes), and incomplete scope (inability to predict subtypes of the voltage-gated channels). We developed a first-of-its-kind PSIONplus^m^ method that performs sequential multi-label prediction of ion channels and their subtypes for both voltage-gated and ligand-gated channels. PSIONplus^m^ sequentially combines the outputs produced by three support vector machine-based models from the PSIONplus predictor and is available as a webserver. Empirical tests show that PSIONplus^m^ outperforms current methods for the multi-label prediction of the ion channel subtypes. This includes the existing single-label methods that are available to the users, a naïve multi-label predictor that combines results produced by multiple single-label methods, and methods that make predictions based on sequence alignment and domain annotations. We also found that the current methods (including PSIONplus^m^) fail to accurately predict a few of the least frequently occurring ion channel subtypes. Thus, new predictors should be developed when a larger quantity of annotated ion channels will be available to train predictive models.

## 1. Introduction

Ion channels are integral membrane proteins that regulate the flow of anions and voltage potential across cellular membranes. They are typically classified into two broad types according to their gating mechanism: voltage-gated [1] vs. ligand-gated [2]. Ion channels can also be categorized based on the type of the passing ions into several subtypes: potassium (K^+^), sodium (Na^+^), calcium (Ca^2+^), and anion channels [1,2,3]. Some of the ion channels are selective to specific ions while others may transport multiple ion types. These channels are associated with a wide range of pathologies including cardiac arrhythmias, epilepsy, hyperthermia, and hyperinsulinism [4] and offer an opportunity to regulate and combat several types of cancer [5,6,7]. Many of the ion channels are considered as potent and promising drug targets [8,9,10,11]. Correspondingly, as many as 15% of the protein drug targets in the human proteome are ion channels [12]. Moreover, over 400 putative ion channels were already identified in human and they are estimated to account for as much as 1% of the protein coding genes in human [11]. The number of the annotated ion channel proteins has grown rapidly in recent years, from about 400 in the mid-1990s to close to 3000 by 2015 [13]. However, thousands of ion channels remain to be identified among the over 175 million of the already sequenced proteins (source: UniProt release 2019_11 [14]).

The practical importance of ion channels as drug targets combined with the need to identify them in the vast collections of unannotated protein sequences motivates the development of computational methods that predict ion channels directly from protein chains. Several such predictors were developed in the last quindecennial. They include three methods that narrowly focus on the prediction of subfamilies of the voltage-gated potassium channels [15,16,17]. We focus on a more generic prediction that identifies both voltage- and ligand-gated channels and which considers several subtypes including potassium, calcium, sodium, and chloride. Six of these predictors were published so far. The first method that was released in 2006, VGIchan, predicts ion channels and four of their subtypes: potassium, sodium, calcium, and chloride [18]. However, VGIchan does not identify the channel type (voltage- vs. ligand-gated). IonchanPred 1.0 that was published in 2011 predicts ion channels, their types and four major subtypes of the voltage-gated ion channels: potassium, sodium, calcium, and anions [19]. This predictor was recently upgraded to a new version, IonchanPred 2.0, which boasts improved predictive performance and which focuses on the same two types of ion channels and the four subtypes of the voltage-gated ion channels [20]. The other three predictors, which include the method by Tiwari and Srivastava [21], the method by Han et al. [22], and PSIONplus [23], address the prediction of ion channels, their types (voltage- vs. ligand-gated) and the four subtypes of the voltage-gated channels (potassium, sodium, calcium, and anions). These predictors use sophisticated machine learning algorithms, such as support vector machines [18,19,20,22,23] and random forest [21,22], and secure relatively good predictive performance [13]. 

A recent survey points to three major drawbacks of the current methods [13]. **First**, only three of the six tools, IonchanPred2.0 [20], VGIchan [18] and PSIONplus [23], are available to the end user. The lack of available implementations renders the other three tools practically unusable. The survey states that the provision of the implementation or webserver should be required at the time the corresponding article is published [13]. **Second**, each of the six current methods is composed of three separate predictive models that make predictions in parallel, i.e., a given input sequence is predicted as ion channel vs. non-ion channel; as voltage- vs. ligand-gated channel; and as one of the four subtypes of the voltage-gated channels. This means that the prediction of channel type must be performed for the known ion channels since the outcomes are limited to only the voltage- vs. ligand-gated channels. Similarly, the prediction of the subtypes must be performed only for the known voltage-gated channels. This type of parallel prediction is inconvenient since end users must run the three models manually one after another. Moreover, the corresponding empirical assessment that was published with these tools is potentially misleading as it also runs the tests in parallel, where the predictors of the channel types (voltage-gated channel subtypes) are evaluated on the already pre-selected ion channels (voltage-gated channels). The more appropriate prediction should be executed sequentially in three steps, where the input protein sequence is first predicted as non-ion channels vs. ion channels, followed by the second step which predicts the channel type for the putative ion channels, and concluding with the prediction of the voltage-gated channel type for the putative voltage-gated channel predicted in the second step. Such sequential test regimes may result in an accumulation of errors along the subsequent steps. A recent comparative survey evaluated the current methods using the sequential regime and concluded that the most accurate PSIONplus offers modest levels of predictive performance [13]. **Third**, the current tools predict a single outcome for each input protein sequence, while in fact some of the channels may transport multiple types of ions. This requires a multi-label prediction where a given method can output multiple ion channel subtypes. The survey reveals that combining outputs generated by multiple current single-label predictors does not offer an accurate solution since they often produce the same subtype or their combined predictions are inaccurate when they differ [13]. Thus, the authors conclude that novel approaches that are specifically designed to make the multi-label predictions are needed. Moreover, the **fourth** problem of the current approaches, which was not covered in the survey, is that they do not consider the subtypes of the ligand-gated channels. They categorize the ion channels into two types: voltage- vs. ligand-gated, and further subdivide the voltage-gated channels into four subtypes: sodium, calcium, potassium and anion carrying [13,19,20,21,22,23]. However, they do not annotate the subtypes of the ligand-gated channels.

We propose a new computational method, PSIONplus^m^, for the sequence-based prediction of ion channels, their types and subtypes that addresses the four abovementioned issues. PSIONplus^m^ builds on the top of arguably the most accurate current method [13], PSIONplus [23]. It performs the prediction in a sequential manner, makes multi-label predictions that allow to identify channels that transport multiple ion types, and covers the subtypes for both the voltage-gated and the ligand-gated channels. Moreover, PSIONplus^m^ is available as a free-to-use and user-friendly webserver at https://yanglab.nankai.edu.cn/PSIONplusm/. The corresponding standalone code can be obtained from https://github.com/cliffgao/PSIONplusm. 

## 2. Materials and Methods 

### 2.1. Benchmark Dataset and Annotation of Ion Channel Types and Subtypes

We used a recently introduced protocol to collect and annotate the benchmark dataset [13]. The ion channels were collected from UniProt [14] by using the gene ontology (GO) [24,25] molecular function annotations. We used the high-quality manually reviewed annotations of the relevant GO terms (“ligand-gated ion channel activity” and “voltage-gated ion channel activity”) and keywords (“sodium”, “potassium”, “calcium”, and “anion”), and/or annotations of UniprotKB keywords (“voltage-gated”, “ligand-gated”, “sodium”, “potassium”, “calcium” and “anion”). We also collected the non-ion channels that cover other types of membrane proteins by using the manually reviewed GO molecular function term “membrane” and cellular component term “membrane“, and by excluding proteins that use keyword “channel” in the GO molecular function annotation. Consistent with the recent comparative review [13], we used such non-ion channels to verify whether the ion channel predictors can accurately differentiate between the ion channels and the other types of membrane proteins. Importantly, we ensured that these ion channels and non-ion channels share low sequence similarity, <30%, with the training datasets of IonchanPred 2.0 and PSIONplus—the two current predictors of ion channel types and subtypes that are available to the end users—to facilitate a robust empirical evaluation of these methods. To this end, we used CD-HIT [26,27] with the sequence identity cut-off 30% to cluster our annotated membrane proteins together with the training datasets of the two predictors. The clusters that include any of the training proteins were deleted and we used the remaining ion channels and non-ion channels to develop the benchmark dataset. This way, the benchmark proteins share <30% similarity with the training proteins, while they may still share higher levels of similarity with other benchmark proteins. Further details can be found in [13]. 

Next, we manually verified and extended the annotations of the ion channels and their types/subtypes for the remaining ion channels. As we discussed in the introduction, the current predictors do not consider the subtypes of the ligand-gated channels and they assume that each channel is categorized into a single subtype. We addressed both problems by annotating each ion channel into one of the two types and one of more of the four subtypes, thus also allowing for multiple subtype annotations. We removed the proteins without complete labels, for which we were not able to identify the type or the subtype. Altogether, we collected 110 ion channels for which we have completed type and subtype annotations. We also included a size-matched set of 111 non-ion channels. These 221 proteins are dissimilar to the training datasets of IonchanPred 2.0 and PSIONplus. We summarize this dataset Table 1. The dataset includes 29 proteins with multiple labels (multiple subtype annotations) and the corresponding average and median cardinality of the labels are 1.32 and 1, respectively. The complete list of the 221 proteins together with the annotations is provided in the Supplementary Materials.

### 2.2. Sequential Multi-Label Prediction 

**Figure 1:**
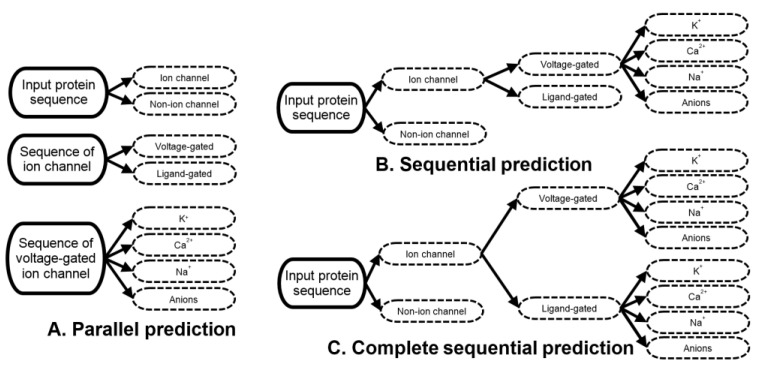
Parallel (**A**), sequential (**B**) and complete sequential (**C**) prediction of the ion channels and their types and subtypes. The solid lines denote inputs while dashed black lines denote putative annotations generated by predictive models.

As we discuss in the introduction, the current methods perform parallel prediction of the single-label ion channels, ion channel types and voltage-gated channel subtypes [13]. In other words, the input protein sequence is independently predicted as ion channel vs. non-ion channel, as voltage- vs. ligand-gated channel, and as one of the four subtypes of the voltage-gated channels. Given that the prediction of the ion channel types and subtypes is limited to the two and four outcomes, respectively, this approach to prediction assumes that the input protein is a known ion channel when being predicted for the channel types, and is a known voltage-gated channel when being predicted for the channel subtype (Figure 1A). These assumptions are impractical when the end users want to predict types and subtypes of the channels for uncharacterized sequences.

A practical way to perform this prediction is to predict the channels, their types and subtypes in three sequential steps [13]. First, the input protein sequences should be predicted as either non-ion channels or ion channels. Second, the putative ion channels predicted in the first step should be processed to predict their types. Third, the channel subtypes should be predicted for the putative voltage-gated channels that were predicted in the second step. The third step is currently limited to the prediction of the subtypes for the voltage-gated channels because this is how the current predictors operate [13,19,20,21,22,23] (Figure 1B). The approach implemented by PSIONplus^m^ extends the sequential prediction to cover the subtypes of the ligand-gated channels (Figure 1C). 

Moreover, in contrast to the current tools that are limited to the prediction of a single channel subtype [13,19,20,21,22,23], PSIONplus^m^ predicts multiple subtypes of the ion channels. This type of prediction can be accomplished in three ways: (1) by combining results produced by multiple current ion channel predictors; (2) by combining multiple new predictors where each individual new predictor targets a specific subtype of channels; and (3) by developing a new single multi-label predictor. As we mention in the introduction, the first approach was recently tested empirically showing relatively poor predictive performance [13]. The second approach was used in a related context to predict residues that interact with multiple types of ligands: DNA, RNA, and proteins [28,29,30,31,32,33,34,35,36]. In this case different predictive models were used to predict residues that bind to specific types of ligands, and these methods were combined together to effectively predict interaction with the multiple types of ligands for the same residue [29,30,35]. However, this architecture is not compatible with the hierarchical nature of the annotations of the ion channels where both types of channels are categorized into the same set of subtypes. This would require the development of eight models, which is prohibitive given the limited amount of the available training data. The third option is to design one predictor that produces multiple outcomes for the same input protein sequence [37]. Several such multi-label models that predict protein and gene functions [38,39,40] and subcellular locations [41,42,43] were released recently. Our design is inspired by the latter approach, where we use a single model to generate multi-label predictions (to cover multiple subtypes of the ion channels) in the sequential manner shown in Figure 1C.

### 2.3. Evaluation of the Predictive Performance

The assessment of predictive performance of the predictors of ion channels and their types and subtypes typically relies on several metrics that include accuracy, sensitivity (also called recall and true positive rate), precision and F_1_ [13,19,20,21,22,23]. 

Given a multi-label dataset *D* that includes |*D*| samples (*p_i_*, *l_i_*) where *i* = 1,…|*D*|, *p_i_* denotes *i*th protein annotated with label(s) *l_i_* ⊆ *L* = {non-ion channel, ligand-gated potassium channel, ligand-gated sodium channel, ligand-gated calcium channel, ligand-gated anion channel, voltage-gated potassium channel, voltage-gated sodium channel, voltage-gated calcium channel, voltage-gated anion channel}, H that is a multi-label predictor where z*_i_* = H(*p_i_*) is the set of labels predicted by H for protein *p_i_*, we use the following set of metrics:

(1) $$\mathrm{Accuracy}=\frac{1}{\left|D\right|}\sum_{i=1}^{\left|D\right|}\frac{\left|l_{i}\cap^{\text{​}}z_{i}\right|}{\left|l_{i}\cup^{\text{​}}z_{i}\right|};\;\mathrm{Precision}=\frac{1}{\left|D\right|}\sum_{i=1}^{\left|D\right|}\frac{\left|l_{i}\cap^{\text{​}}z_{i}\right|}{\left|z_{i}\right|};\;\mathrm{Recall}=\frac{1}{\left|D\right|}\sum_{i=1}^{\left|D\right|}\frac{\left|l_{i}\cap^{\text{​}}z_{i}\right|}{\left|l_{i}\right|};\;F_{1}=\frac{2Recall*Precision}{Recall+Precision}$$

### 2.4. Architecture of the PSIONplus^m^ Predictor

**Figure 2:**
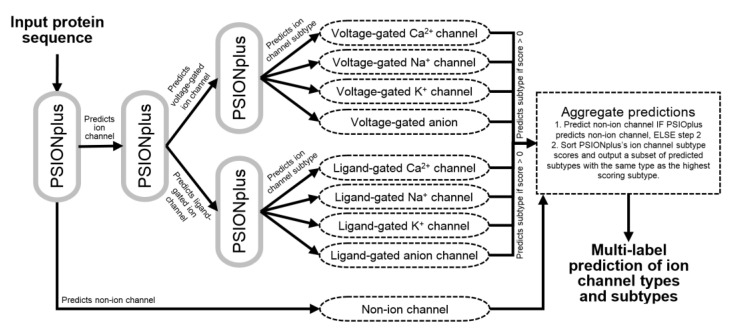
Flowchart of the prediction protocol implemented by PSIONplus^m^.

We design PSIONplus^m^ by extending the currently most accurate method [13], PSIONplus [23], to make sequential multi-label predictions that cover the four subtypes of ligand-gated and voltage-gated channels. PSIONplus consists of three support vector machine (SVM)-based models: PSION_ION_ that predicts ion channels vs. non-ion channels; PSION_VLG_ that predicts voltage-gated ion channels vs. ligand-gated ion channels, and PSION_VGS_ that predicts the four subtypes of the voltage-gated ion channels. We use these three models sequentially by passing the resulting predictions into the subsequent models (Figure 2). These predictions are aggregated to produce the multi-label outputs as follows. PSIONplus^m^ predicts the non-ion channel if PSIONplus predicts this label. Otherwise, we sort the PSIONplus’s ion channel subtype scores (which are computed by multiplying the propensities generated by the PSION_VLG_ and PSION_VGS_ models) and we output a subset of the predicted subtypes with the same type as the highest scoring subtype that has non-zero channel subtype scores. This means that we use the PSION_VGS_’s predictions of the four subtypes to predict the subtypes of both the voltage-gate and the ligand-gated channels. This is possible since the PSION_VGS_ model works in parallel to the PSION_VLG_ model. We limit our predictions to the subtypes with the non-zero scores to eliminate subtypes that were excluded by PSIONplus. We further limit the predicted subtypes to the same type of the channel (selected as the type that secures the highest score) given that empirical tests on the benchmark dataset reveal that this leads to favorable predictive performance. In other words, we start with the highest scoring subtype predicted by PSIONplus and we add lower ranked (according to the sort) subtype only if it has the same channel type and different subtype. The corresponding architecture of PSIONplus^m^ is shown in Figure 2. We emphasize that we did not re-train the original PSIONplus model and that the entire predictive process is parameterless. Correspondingly we did not need a training dataset to develop the PSIONplus^m^, other than the training dataset that was originally used to train PSIONplus. 

## 3. Results

### 3.1. Ion Channels and Their Types and Subtypes Are Hard to Predict Directly from the Sequence

A recent study showed that predictions that rely on sequence similarity computed with BLAST are inferior to the results generated by PSIONplus [23]. These predictions are based on the pairwise alignments of a given test sequence against the sequences from the training dataset of PSIONplus. This way, both PSIONplus and the alignment-based predictor rely on the same set of training proteins, i.e., annotated ion channels. The alignment-based predictor transfers the ion channel annotations from the most similar training sequence given that it is sufficiently similar, otherwise the non-ion channel label is predicted. The minimal level of similarity was empirically optimized to maximize the predictive performance. The underlying reason for the lower predictive performance of the BLAST-based predictions is the fact that the test proteins share low similarity with the training proteins, simulating the expected scenario where novel ion channels (i.e., channels that are dissimilar to the currently known ion channels) are being predicted.

We consider an alternative approach that predicts ion channels and their types/subtypes directly from the sequence. We exploit evolutionary relationships between the known/training ion channels and the test proteins that we detect via presence of the same domains. First, we use the protocol from Section 2.1 to annotate the ion channels and their types/subtypes for the training dataset of PSIONplus [23]. We remove the training proteins that we could not find in the current version of UniProt and those for which the annotation could not be completed, i.e., the subtype information is unavailable. We managed to annotate 466 proteins from the original set of 598 training proteins. We note that this dataset shares low similarity to our benchmark dataset. Second, we collect the Pfam domains [44] for the 466 training proteins and the 221 proteins from our benchmark dataset. Table 2 summarizes this step by listing how many of these proteins have at least one Pfam domain. We find that between 77% (for the voltage gated anion channels in the benchmark dataset) and 100% of proteins (for all ligand gated channels in both datasets, and the voltage gated sodium and potassium channels in the training dataset) have annotated domains. The average fraction of proteins that have Pfam domain, which is computed across the nine labels and both datasets, equals 96.7%. Third, we make predictions using the Pfam domains, given that they are available for the significant majority of the training and test proteins. For each of the eight ion channel subtypes we collect the domains that are present in the corresponding training proteins, creating eight training domain sets. A test protein is predicted with a given label (ion channel type and subtype) if at least one of its domains is present in the corresponding training domain set. The test proteins that lack domain annotations and that have domains that do not overlap with any of the eight training domain sets are predicted as the non-ion channels. This procedure generates the multi-label predictions since some of the test proteins may have domains that are present in multiple training domain sets.

Table 2 summarizes the results generated by the above domain-based predictor. The predictive performance is quantified with an average of the correct prediction rates over proteins with a given label. The rate is computed as the number of correctly predicted labels divided by the number of all predicted labels for a given benchmark sequence. We observe that Pfam domains are semi-accurately predicted only in the voltage-gated sodium channels and the ligand-gated sodium and potassium channels. Several channel types, such as the voltage-gated calcium and anion channels and the ligand gated calcium and anion channels, are poorly predicted. This is because these channels in the benchmark dataset rarely share domains with the channels in the training dataset. We also assess the predictive performance of the overall multi-label predictions on the entire benchmark dataset. The corresponding accuracy = 19.8, precision = 20.1, recall = 26.5, and F_1_ = 22.8. To compare, a random predictor, which is computed by shuffling the actual labels (annotations of ion channels and their types and subtypes) among the benchmark proteins, secures accuracy = 30.6, precision = 31.6, recall = 31.6, and F_1_ = 31.6. This experiment reveals that the domain-based predictor does not offer a viable solution for the prediction of ion channels and their types/subtypes. The underlying reason why neither the alignment-based nor the domain-based approaches provide accurate results stems from the low numbers of the currently know channels that do not cover a much larger and likely more diverse set of ion channels sequences that await annotation, which we represent here by the benchmark dataset. The machine learning predictors, such as PSIONplus, IonchanPred2.0 and VGIchan, offer a more viable alternative, as shown in the recent studies [13,23]. Instead of using sequence similarity or presence of common domains, they exploit similarity in the multi-dimensional space defined by physiochemical characteristics of the sequences. For instance, PSIONplus utilizes information about the hydrophilicity, hydrophobicity, polarity, transfer free energy and putative secondary structure, solvent accessibility, intrinsic disorder to make the predictions [23].

### 3.2. Comparative Assessment of PSIONplus^m^

**Figure 3:**
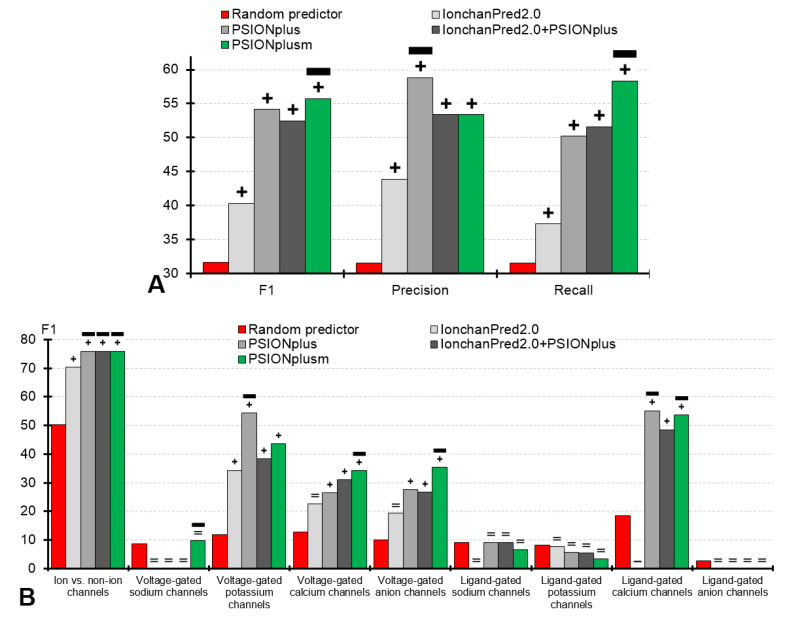
Predictive performance for the sequential prediction with PSIONplus^m^, IonchanPred2.0, PSIONplus, combination of predictions from IonchanPred2.0 and PSIONplus (IonchanPred2.0+PSIONplus) and the random predictor (implemented by shuffling of actual labels) on the benchmark dataset. Panel (**A**) summarizes the values of F_1_, precision, and recall metrics for the multi-label prediction of channels and their types. Panel (**B**) shows F_1_ values for individual outcomes including the prediction of ion channels and their 8 types/subtypes. Annotations above the bars denote the statistical significance of the differences between the random prediction and each of the four predictors, where +, −, and = denote that a given predictor is significantly better, significantly worse, and not significantly different to the random predictor. The thick horizontal black lines identify the ion channel predictors that outperform the random predictor and which are statistically significantly better than the other channel predictors for a given label. We assume that the difference is significant when *p*-value < 0.001. Calculation of significance is explained in the footnote in Table 3.

We compare PSIONplus^m^ with IonchanPred 2.0 and PSIONplus, which are the only two other methods that predict ion channel types and subtypes and that are available to the end users. We also produce an alternative version of the multi-label predictor by combining the outputs from IonchanPred2.0 and PSIONplus; this approach is denoted as IonchanPred2.0+PSIONplus. Moreover, we contrast results produced by these four methods against the random predictor implemented by shuffling the actual labels (annotations of ion channels and their types and subtypes) in the benchmark dataset. We assess the predictive performance of the sequential prediction on the benchmark dataset. This dataset is dissimilar (<30% sequence similarity) to the training datasets of PSIONplus, IonchanPred2.0, and PSIONplus^m^; the latter method relies on the same training dataset as PSIONplus. Table 3 provides a comprehensive set of metrics for the overall multi-label prediction (the top row), and for the prediction of the nine individual outcomes (ion channels and the eight subtypes of the ligand- and voltage-gated channels). Figure 3 summarizes the key metrics for the overall prediction (in panel A) and for the individual outcomes (panel B).

#### 3.2.1. Assessment of the Overall Multi-label Prediction of the Ion Channels and Their Types and Subtypes

Our empirical analysis reveals that the four considered here predictors (PSIONplus^m^, IonchanPred 2.0, PSIONplus, and IonchanPred2.0+PSIONplus) secure statistically significantly better F_1_ values when compared against the random predictor (*p*-value < 0.001; Figure 3A). The same significant improvements are present for the precision, recall and accuracy (*p*-value < 0.001; Figure 3A and the top row in Table 3). This suggests that these methods provide useful information to identify ion channels, their types and subtypes.

The two single-label predictors, IonchanPred2.0 and PSIONplus, are outperformed by the multi-label PSIONplus^m^. Table 3 (top row) reveals that PSIONplus^m^ secures F_1_ = 55.7% compared to 54.1% for PSIONplus and 40.3% for IonchanPred2.0; these improvements are statistically significant (*p*-value < 0.001). While PSIONplus offers higher precision than PSIONplus^m^ (58.8% vs. 53.4%), the new predictor has significantly higher recall (58.3% vs. 50.2%; *p*-value < 0.001); see Figure 3A. This is because PSIONplus^m^’s multi-label output covers more ion channel subtypes, resulting in the improved recall. The PSIONplus^m^’s precision of 53.4% and recall of 58.3% mean that 53.4% of its predictions are correct and that it correctly predicts 58.3% of the native labels. These are relatively good levels of predictive performance given that this problem concerns nine labels/outcomes and that the random predictor provides precision = 31.6% and recall = 31.6%. In short, our empirical analysis shows that the first-of-its-kind multi-label PSIONplus^m^ provides useful predictions that are significantly better than the outputs produced by the current single-label predictors.

We also evaluate an alternative multi-label predictor generated as a simple union of the results produced by the two single-label predictors (IonchanPred2.0+PSIONplus). This approach is significantly outperformed by PSIONplus^m^ in F_1_ (55.7% vs. 52.5%; *p*-value < 0.001) and recall (58.3% vs. 51.6%; *p*-value < 0.001), while maintaining the same precision of 53.4% (Figure 3A and the top row in Table 3). Moreover, this simple multi-label predictor has lower F_1_, lower precision, and a slightly higher recall when contrasted with PSIONplus. Overall, we conclude that this alternative multi-label predictor does not produce improvements compared to the original single-label predictors, which is in agreement with the observations in [13].

#### 3.2.2. Assessment of the Prediction of the Ion Channels

The metrics for the prediction of the ion channels are summarized in the first set of bars in Figure 3B and in the second row in Table 3. We show that the four predictors (PSIONplus^m^, IonchanPred 2.0, PSIONplus, and IonchanPred2.0+PSIONplus) are significantly better than the random predictor in F_1_, precision, and recall (*p*-value < 0.001). Moreover, PSIONplus^m^ and PSIONplus have equivalent levels of predictive performance while offering significant improvements over IonchanPred 2.0 (*p*-value < 0.001) with F_1_ = 76.0% vs. 70.4% and recall = 71.2% vs. 62.2%. The difference between PSIONplus and IonchanPred 2.0 is consistent with the results reported in [13]. We conclude that both PSIONplus^m^ and PSIONplus accurately identify ion channels from the protein sequences. 

#### 3.2.3. Assessment of the Prediction of the Ion Channel Subtypes

The metrics for the prediction of the ion channel subtypes are summarized in Figure 3B (except for the first set of bars) and in the third and subsequent rows in Table 3. 

First, we observe that all considered methods (PSIONplus^m^, IonchanPred 2.0, PSIONplus, and IonchanPred2.0+PSIONplus) fail to accurately predict the following four subtypes of the ion channels: voltage-gated sodium channels, ligand-gated sodium channels, ligand-gated potassium channels, and ligand-gated anion channels. Their predictive performance is equivalent to that of the random predictor (Table 3). A potential reason could be related to the fact that these subtypes of ion channels have the lowest counts in the benchmark dataset (Table 1), and likely also in the datasets used to train the current predictors. Moreover, for PSIONplus^m^, another reason is likely related to the fact that the scores from PSIONplus that we use to implement the predictions of the subtypes of the ligand-gated channels in PSIONplus^m^ were originally optimized to predict the subtypes of the voltage-gated channels. 

Second, only PSIONplus^m^ provides consistently and significantly higher predictive performance measured with F_1_, precision, and recall when compared with the random predictor for the other four subtypes of the ion channels: voltage-gated potassium channels, voltage-gated calcium channels, voltage-gated anion channels, and ligand-gated calcium channels (*p*-value < 0.001; Table 3). Other predictors offer a more spotty performance, where IonchanPred 2.0 improves only for the voltage-gated potassium channels, PSIONplus for the voltage-gated potassium channels and the ligand-gated calcium channels, and the combination method (IonchanPred2.0+PSIONplus) for the voltage-gated potassium channels, voltage-gated calcium channels and ligand-gated calcium channels. 

Third, PSIONplus^m^ is significantly better (based on the F_1_ value) than the other three methods (IonchanPred 2.0, PSIONplus, and IonchanPred2.0+PSIONplus) for the prediction of the voltage-gated calcium channels and voltage-gated anion channels (*p*-value < 0.001). Moreover, both PSIONplus^m^ and PSIONplus are significantly better than IonchanPred2.0 for the prediction of the ligand-gated calcium channels (*p*-value < 0.001), while PSIONplus is the best for the prediction of the voltage-gated potassium channels (*p*-value < 0.001) (Figure 3B).

Overall, we conclude that PSIONplus^m^ is the best alternative among the methods considered here including IonchanPred 2.0, PSIONplus, the domain-based predictor, and the sequence alignment-based approach, given that the latter method was empirically shown to be outperformed by PSIONplus [23]. Moreover, our empirical results suggest that new multi-label predictors are needed, particularly for the prediction of the voltage-gated sodium channels, ligand-gated sodium channels, ligand-gated potassium channels, and ligand-gated anion channels. The development of these methods may require a more comprehensive data curation to acquire larger sets of these subtypes of the ion channels.

## 4. PSIONplus^m^ Webserver

**Figure 4:**
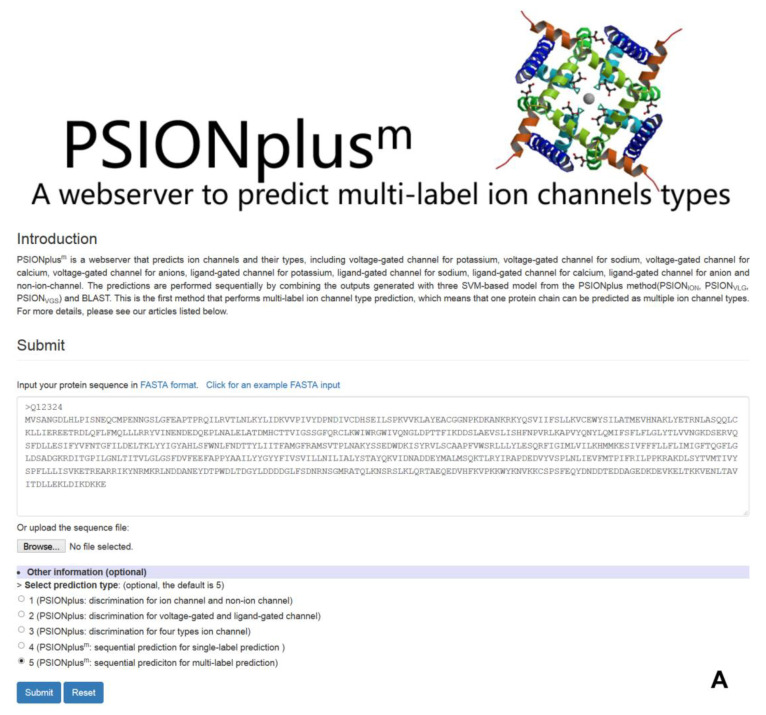

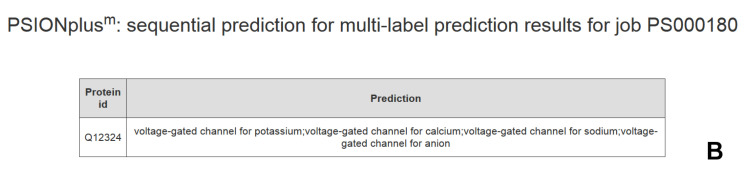
Web interface of the PSIONplus^m^ webserver at https://yanglab.nankai.edu.cn/PSIONplusm/. Panel (**A**) shows the main page of the webserver while panel (**B**) shows an example output produced by the webserver.

PSIONplus^m^ predictor is freely available to the end users as a webserver located at https://yanglab.nankai.edu.cn/PSIONplusm/. Figure 4 shows the web interface of this webserver. 

PSIONplus^m^ webserver needs the FASTA-formatted protein sequence as the only input. The webserver offers an option to select one of the five available predictive models (Figure 4A): (1) PSIONplus for the prediction of ion channel vs. non-ion channel; (2) PSIONplus for the prediction of voltage-gated vs. ligand-gated ion channels (assuming that the input sequence is an ion channel); (3) PSIONplus for the prediction of the four subtypes of the voltage-gated ion channels (assuming that the input sequence is a voltage-gated ion channel); (4) PSIONplus for the sequential single-label prediction of the ion channels and ion channel types and subtypes; and finally (5) PSIONplus^m^ for the sequential multi-label prediction of the ion channels and ion channel types and subtypes. By default, the webserver performs the predictions using the PSIONplus^m^ model.

The computations are performed on the server side and the results are returned to the user in the browser window (Figure 4B). The entire prediction process takes about 2 to 3 min.

## 5. Discussion and Conclusions

The prediction of the ion channels and their types/subtypes offers a viable and efficient way to identify putative ion channels in the vast databases of protein sequences. While the current methods were shown to offer reasonably accurate predictions [13,20,22,23], they suffer a number of drawbacks including the lack of availability, parallel prediction modes, an inability to predict multiple channel subtypes (they perform single-label prediction), and a lack of support for the prediction of the subtypes of the voltage-gated channels. Our new predictor, PSIONplus^m^, addresses these issues by performing sequential multi-label prediction of ion channels and their subtypes for both voltage-gated and ligand-gated ion channels. PSIONplus^m^ is freely available as a convenient to use webserver located at https://yanglab.nankai.edu.cn/PSIONplusm/. We also provide the standalone version of this predictor via its GitHub page at https://github.com/cliffgao/PSIONplusm.

We test and empirically compare PSIONplus^m^ with the current methods that are available to the end users (IonchanPred2.0, PSIONplus, a simple multi-label approach that combines results from these two methods: IonchanPred2.0+PSIONplus, and the domain-based approach) on a new benchmark dataset that shares low similarity with the training datasets used to build these predictors. We demonstrate that PSIONplus^m^ significantly outperforms the other methods in the overall test that considers multi-label prediction of all channel subtypes. We also show that the alternative multi-label predictor that combines results produced by IonchanPred2.0 and PSIONplus underperforms compared to the single-label PSIONplus and the multi-label PSIONplus^m^. Further analysis reveals that PSIONplus^m^ produces the most accurate predictions for the voltage-gated calcium channels and the voltage-gated anion channels, and together with PSIONplus for the prediction of the ion channels and the ligand-gated calcium channels. 

However, we also found that all considered methods (PSIONplus^m^, IonchanPred 2.0, and PSIONplus) have difficulty with the prediction of the voltage-gated sodium channels, ligand-gated sodium channels, ligand-gated potassium channels, and ligand-gated anion channels. Their predictive performance for these ion channel subtypes is equivalent to a random predictor. This is likely connected with the fact that these channel subtypes are the least frequent in the current training and benchmark datasets. 

We recommend that novel multi-label predictors should be built to provide improved predictions, particularly for the currently poorly predicted ion channel subtypes. There are at least two potential avenues for the development of the future methods. First, while PSIONplus^m^ combines multiple single-label SVM models, it would be beneficial to investigate the application of multi-label models [37]. Such approaches were recently applied for several related prediction problems including enzyme type [45], protein functions [38], and subcellular locations prediction [42,43]. Second, new and larger training datasets should be developed as new annotations of ion channels become available in the future. In other words, the efforts to develop novel methods will require a careful and comprehensive curation of new datasets that include larger numbers of proteins that uniformly cover different channel subtypes and that provide a comprehensive representation for the non-channel proteins.

## Figures and Tables

**Table 1:** Summary of the benchmark multi-label dataset.

Protein Type	Ion Channel Type	Ion Channel Subtype	Number of Proteins
Ion channels	Voltage-gated	Sodium (Na^+^)	19
		Potassium (K^+^)	26
		Calcium (Ca^2+^)	28
		Anions	22
	Ligand-gated	Sodium (Na^+^)	20
		Potassium (K^+^)	18
		Calcium (Ca^2+^)	41
		Anions	6
Non-ion channels (other types of membrane proteins)			111
Total number of proteins			221

**Table 2:** Coverage by Pfam domains and the average rate of correct predictions for the domain-based prediction of ion channels and their subtypes. The right-most column is the average of the correct prediction rates across proteins with a given label, where the rate is computed as the number of correctly predicted labels divided by the number of all predicted labels.

Prediction Target/Label	% of Proteins with at Least One Pfam Domain		Average Rate of Correct Predictions on Benchmark Dataset
	PSIONplus Training Dataset	Benchmark Dataset	
Non-ion channels	93.0%	95.5%	92.8%
Voltage-gated sodium channels	100.0%	94.7%	58.9%
Voltage-gated potassium channels	100.0%	96.2%	49.4%
Voltage-gated calcium channels	96.6%	96.4%	14.3%
Voltage-gated anion channels	90.9%	77.3%	13.6%
Ligand-gated sodium channels	100.0%	100.0%	64.8%
Ligand-gated potassium channels	100.0%	100.0%	71.7%
Ligand-gated calcium channels	100.0%	100.0%	4.9%
Ligand-gated anion channels	100.0%	100.0%	0.0%

**Table 3:** Evaluation of the sequential prediction of ion channels and their subtypes on the benchmark dataset. The random predictor is implemented by shuffling the actual labels; we report the average based on 1000 repetitions. The IonchannelPred2.0+PSIONplus is a multi-label prediction that combines outputs generated by these two methods. The best values for each row (a given quality index and outcome) are shown in bold font.

Prediction Target/Label	Measure	Predictors				
		Random	IonchanPred2.0	PSIONplus	IonchanPred2.0+ PSIONplus	PSIONplus^m^
Overall (multi-label prediction of ion channels and their types)	F_1_	31.6	40.3 ^+/− a^	54.1 ^+/−^	52.5 ^+/−^	**55.7 ^+^**
	Accuracy	30.6	37.3 ^+/−^	**50.2 ^+/+^**	46.6 ^+/−^	47.1 ^+^
	Precision	31.6	43.9 ^+/−^	**58.8 ^+/+^**	53.4 ^+/=^	53.4 ^+^
	Recall	31.6	37.3 ^+/−^	50.2 ^+/−^	51.6 ^+/−^	**58.3 ^+^**
Ion vs. Non-ion channels	F_1_	50.2	70.4 ^+/−^	**76.0** ^+/=^	**76.0** ^+/=^	**76.0** ^+^
	Precision	0.2	81.2 ^+/=^	**81.4** ^+/=^	**81.4** ^+/=^	**81.4** ^+^
	Recall	50.2	62.2 ^+/−^	**71.2** ^+/=^	**71.2** ^+/=^	**71.2** ^+^
Voltage-gated sodium channels	F_1_	8.6	0.0 ^=/-^	0.0 ^=/-^	0.0 ^=/-^	**9.7** ^=^
	Precision	**8.6**	0.0 ^=/-^	0.0 ^=/-^	0.0 ^=/-^	7.0 ^=^
	Recall	8.6	0.0 ^=/-^	0.0 ^=/-^	0.0 ^=/-^	**15.8** ^=^
Voltage-gated potassium channels	F_1_	11.8	34.3 ^+/−^	**54.3** ^+/+^	38.3 ^+/−^	43.6 ^+^
	Precision	11.8	22.8 ^=/-^	**40.0** ^+/+^	24.5 ^+/−^	29.3 ^+^
	Recall	11.8	69.2 ^+/−^	84.6 ^+/=^	**88.5** ^+/+^	84.6 ^+^
Voltage-gated calcium channels	F_1_	12.7	22.6 ^=/-^	26.4 ^+/−^	31.0 ^+/−^	**34.2** ^+^
	Precision	12.7	24.0 ^=/=^	**28.0** ^+/=^	25.6 ^+/−^	27.1 ^+^
	Recall	12.7	21.4 ^=/-^	25.0 ^=/-^	39.3 ^+/−^	**46.4** ^+^
Voltage-gated anion channels	F_1_	10.0	19.4 ^=/-^	27.6 ^+/−^	26.7 ^+/−^	**35.3** ^+^
	Precision	10.0	33.3 ^+/=^	**57.1** ^+/+^	50.0 ^+/+^	26.1 ^+^
	Recall	10.0	13.6 ^=/-^	18.2 ^=/-^	18.2 ^=/-^	**54.5** ^+^
Ligand-gated sodium channels	F_1_	9.0	0.0 ^=/=^	**9.1** ^=/=^	**9.1** ^=/=^	6.6 ^=^
	Precision	9.0	0.0 ^=/-^	**50.0** ^+/+^	**50.0** ^+/+^	4.9 ^=^
	Recall	9.0	0.0 ^=/-^	5.0 ^=/-^	5.0 ^=/-^	**10.0** ^=^
Ligand-gated potassium channels	F_1_	**8.1**	7.7 ^=/+^	5.7 ^=/+^	5.4 ^=/+^	3.4 ^=^
	Precision	8.1	**12.5** ^=/+^	5.9 ^=/+^	5.3 ^=/+^	2.4 ^=^
	Recall	**8.1**	5.6 ^=/=^	5.6 ^=/=^	5.6 ^=/=^	5.6 ^=^
Ligand-gated calcium channels	F_1_	18.5	0.0 ^−/−^	**55.2** ^+/=^	48.5 ^+/−^	53.7 ^+^
	Precision	18.5	0.0 ^−/−^	**94.1** ^+/+^	64.0 ^+/+^	53.7 ^+^
	Recall	18.5	0.0 ^−/−^	39.0 ^+/−^	39.0 ^+/−^	**53.7** ^+^
Ligand-gated anion channels	F_1_	**2.7**	0.0 ^=/=^	0.0 ^=/=^	0.0 ^=/=^	0.0 ^=^
	Precision	**2.7**	0.0 ^=/=^	0.0 ^=/=^	0.0 ^=/=^	0.0 ^=^
	Recall	**2.7**	0.0 ^=/=^	0.0 ^=/=^	0.0 ^=/=^	0.0 ^=^

^a^ we report statistical significance of the differences between the random prediction and each of the four predictors of ion channels, and also between the PSIONplus^m^ and the other three predictors of ion channels, where +, −, and = denote that a given predictor is significantly, significantly worse, and not significantly different to the other method. For instance, +/− for the overall prediction and F_1_ for IonchannelPred 2.0 means F_1_ of IonchannelPred 2.0 is significantly better than the F_1_ of the random predictor and significantly worse than the F_1_ of PSIONplus^m^. Comparison to the random predictor is based on 99.9% confidence interval over the 1000 repetitions (*p*-value < 0.001). Comparison with PSIONplus^m^ is based on 100 tests on randomly selected 50% of the benchmark proteins to ensure that the differences are robust across a diverse set of datasets. The significance was measured using paired *t*-test and the differences are assumed significant if *p*-value < 0.001.

## Supplementary Materials

The following are available online at https://www.mdpi.com/2218-273X/10/6/876/s1, The Supplementary Materials that include the benchmark dataset are available online.
