# Supplementary material for: PSIONplusm Server for Accurate Multi-Label Prediction of Ion Channels and Their Types
Source: Biomolecules. 2020 Jun 7;10(6):876. doi: 10.3390/biom10060876 (PMC7355608; doi:10.3390/biom10060876)
Supplement: Supplementary file 1 [file biomolecules-10-00876-s001.pdf]

biomolecules

MDPI

Supplement for "PSIONplus^{m} server for accurate multi-label prediction of ion channels and their types"

Jianzhao Gao [1], Hong Wei [1] , Alberto Cano [2] and Lukasz Kurgan [2,*]

[1] School of Mathematical Sciences and LPMC, Nankai University, Tianjin 300071, China; gaojz@nankai.edu.cn (J.G.); weihong96@mail.nankai.edu.cn (H.W.)
[2] Department of Computer Science, Virginia Commonwealth University, Richmond, VA 23284, USA; acano@vcu.edu (A.C.); lkurgan@vcu.edu (L.K.)
* Correspondence: lkurgan@vcu.edu; Tel.: 804-827-3986

The benchmark dataset with 221 proteins. Each protein is represented using a line that starts with > followed by the UniProt accession number, | separator, and the complete list of annotations/labels separated by semicolon. To accommodate for the changes in UniProt, the sequence of these 221 proteins can be obtained on the website of the PSIONplus^{m} webserver at https://yanglab.nankai.edu.cn/PSIONplusm/benchmark/.

>A2AGL3|ionchannel-ligand-calcium

>A8XP97|ionchannel-voltage-calcium

>B0LPN4|ionchannel-ligand-calcium

>B2ZXD5|ionchannel-voltage-anion

>B7ZAQ6|ionchannel-voltage-anion

>B8AFI8|ionchannel-voltage-anion

>D3Z291|ionchannel-voltage-calcium

>E9PTA2|ionchannel-ligand-calcium;ionchannel-ligand-sodium

>E9PZQ0|ionchannel-voltage-calcium;ionchannel-ligand-calcium

>E9Q401|ionchannel-ligand-calcium

>F1LMY4|ionchannel-voltage-calcium;ionchannel-ligand-calcium

>G5ECJ0|ionchannel-ligand-sodium;ionchannel-ligand-potassium

>H2LRU7|ionchannel-voltage-calcium;ionchannel-voltage-potassium

>O00476|ionchannel-voltage-anion

>O01635|ionchannel-ligand-sodium

>O35245|ionchannel-voltage-calcium;ionchannel-voltage-potassium;ionchannel-voltage-sodium

>O35433|ionchannel-ligand-calcium

>O60741|ionchannel-voltage-potassium;ionchannel-voltage-sodium;ionchannel-ligand-potassium;ionchannel-ligand-sodium

>O70507|ionchannel-voltage-potassium;ionchannel-voltage-sodium;ionchannel-ligand-potassium;ionchannel-ligand-sodium

>O75762|ionchannel-ligand-calcium

>O82226|ionchannel-voltage-potassium;ionchannel-ligand-potassium

>O88703|ionchannel-voltage-potassium;ionchannel-voltage-sodium;ionchannel-ligand-potassium;ionchannel-ligand-sodium

>O88704|ionchannel-voltage-potassium;ionchannel-voltage-sodium;ionchannel-ligand-potassium;ionchannel-ligand-sodium

>O88705|ionchannel-voltage-potassium;ionchannel-voltage-sodium;ionchannel-ligand-potassium;ionchannel-ligand-sodium

>O94759|ionchannel-ligand-calcium;ionchannel-ligand-sodium

>P06756|ionchannel-voltage-calcium

>P08133|ionchannel-ligand-calcium

>P0CG08|ionchannel-voltage-anion

>P11716|ionchannel-voltage-calcium;ionchannel-ligand-calcium

>P11881|ionchannel-ligand-calcium

>P14824|ionchannel-ligand-calcium

>P16960|ionchannel-voltage-calcium;ionchannel-ligand-calcium

>P17785|ionchannel-voltage-calcium

>P21817|ionchannel-voltage-calcium;ionchannel-ligand-calcium

>P29993|ionchannel-ligand-calcium

>P29994|ionchannel-ligand-calcium
>P29995|ionchannel-ligand-calcium
>P30957|ionchannel-ligand-calcium
>P34374|ionchannel-voltage-calcium
>P43406|ionchannel-voltage-calcium
>P48037|ionchannel-ligand-calcium
>P48613|ionchannel-voltage-sodium
>P60300|ionchannel-voltage-anion
>P70227|ionchannel-ligand-calcium
>P83094|ionchannel-ligand-calcium
>Q09428|ionchannel-voltage-potassium
>Q0JKV1|ionchannel-voltage-potassium
>Q12324|ionchannel-voltage-calcium;ionchannel-voltage-potassium;ionchannel-voltage-sodium
>Q13563|ionchannel-voltage-calcium;ionchannel-voltage-potassium;ionchannel-voltage-sodium
>Q13796|ionchannel-ligand-sodium
>Q14571|ionchannel-ligand-calcium
>Q14573|ionchannel-ligand-calcium
>Q14643|ionchannel-ligand-calcium
>Q14644|ionchannel-ligand-calcium
>Q15413|ionchannel-ligand-calcium
>Q18593|ionchannel-voltage-calcium
>Q24498|ionchannel-ligand-calcium
>Q3TXX4|ionchannel-ligand-anion
>Q4GZT3|ionchannel-voltage-calcium;ionchannel-voltage-potassium;ionchannel-voltage-sodium
>Q4KMQ2|ionchannel-voltage-calcium;ionchannel-voltage-anion
>Q54C67|ionchannel-voltage-anion
>Q54LQ4|ionchannel-voltage-anion
>Q5BIM9|ionchannel-voltage-anion
>Q5F448|ionchannel-voltage-anion
>Q5XXA6|ionchannel-voltage-calcium;ionchannel-voltage-anion
>Q62634|ionchannel-ligand-anion
>Q63269|ionchannel-ligand-calcium
>Q697L1|ionchannel-ligand-calcium
>Q6ID99|ionchannel-voltage-anion
>Q6IVV8|ionchannel-voltage-calcium;ionchannel-voltage-potassium
>Q6K965|ionchannel-voltage-anion
>Q6P9J9|ionchannel-voltage-calcium;ionchannel-voltage-anion
>Q6Q473|ionchannel-ligand-anion
>Q6R5A3|ionchannel-ligand-calcium
>Q6RI86|ionchannel-voltage-calcium
>Q704Y3|ionchannel-ligand-calcium;ionchannel-ligand-anion
>Q7TN37|ionchannel-voltage-calcium
>Q7XT08|ionchannel-voltage-potassium
>Q7Z020|ionchannel-ligand-calcium
>Q8BG22|ionchannel-ligand-anion
>Q8BHY3|ionchannel-voltage-anion;ionchannel-voltage-calcium
>Q8BLA8|ionchannel-voltage-calcium
>Q8BS95|ionchannel-voltage-anion
>Q8IU99|ionchannel-voltage-calcium
>Q8NER1|ionchannel-ligand-calcium;ionchannel-ligand-anion
>Q8R4P4|ionchannel-voltage-calcium
>Q8R4P5|ionchannel-voltage-calcium
>Q8WN95|ionchannel-ligand-calcium
>Q91YD4|ionchannel-ligand-calcium;ionchannel-ligand-sodium
>Q92736|ionchannel-ligand-calcium

>Q9FLV9|ionchannel-voltage-anion
>Q9FPG2|ionchannel-voltage-anion
>Q9JKA7|ionchannel-voltage-potassium;ionchannel-voltage-sodium;ionchannel-ligand-potassium;ionchannel-ligand-sodium
>Q9JKA8|ionchannel-voltage-potassium;ionchannel-voltage-sodium;ionchannel-ligand-potassium;ionchannel-ligand-sodium
>Q9JKA9|ionchannel-voltage-potassium;ionchannel-voltage-sodium;ionchannel-ligand-potassium;ionchannel-ligand-sodium
>Q9JKB0|ionchannel-voltage-potassium;ionchannel-voltage-sodium;ionchannel-ligand-potassium;ionchannel-ligand-sodium
>Q9LD83|ionchannel-voltage-anion
>Q9M0A4|ionchannel-voltage-potassium;ionchannel-ligand-potassium
>Q9M2D2|ionchannel-voltage-anion
>Q9MZS1|ionchannel-voltage-potassium;ionchannel-voltage-sodium;ionchannel-ligand-potassium;ionchannel-ligand-sodium
>Q9NA13|ionchannel-ligand-calcium
>Q9P1Z3|ionchannel-voltage-potassium;ionchannel-voltage-sodium;ionchannel-ligand-potassium;ionchannel-ligand-sodium
>Q9S9N5|ionchannel-voltage-potassium;ionchannel-ligand-potassium
>Q9SM57|ionchannel-voltage-anion
>Q9TS33|ionchannel-ligand-calcium
>Q9TV66|ionchannel-voltage-potassium;ionchannel-voltage-sodium;ionchannel-ligand-potassium;ionchannel-ligand-sodium
>Q9UL51|ionchannel-voltage-potassium;ionchannel-voltage-sodium;ionchannel-ligand-potassium;ionchannel-ligand-sodium
>Q9VNB5|ionchannel-ligand-calcium
>Q9Y3Q4|ionchannel-voltage-potassium;ionchannel-voltage-sodium;ionchannel-ligand-potassium;ionchannel-ligand-sodium
>Q9Z329|ionchannel-ligand-calcium
>Q9Y2T6|non_ion
>Q05320|non_ion
>P0A924|non_ion
>Q1ZXS5|non_ion
>Q12851|non_ion
>P40710|non_ion
>Q54HI2|non_ion
>Q8TBE7|non_ion
>O05493|non_ion
>P56159|non_ion
>P39453|non_ion
>O94661|non_ion
>P78768|non_ion
>P07207|non_ion
>Q7JQ32|non_ion
>P0AF16|non_ion
>Q06067|non_ion
>Q9BYB0|non_ion
>P48284|non_ion
>Q7Z699|non_ion
>O81770|non_ion
>P16499|non_ion
>Q6ZMZ3|non_ion
>Q03600|non_ion
>Q7TP54|non_ion
>Q9CWP4|non_ion
>Q9LYU7|non_ion
>Q8IWE4|non_ion
>Q9FGP9|non_ion
>P32710|non_ion
>Q9ERB0|non_ion
>Q9FNR3|non_ion
>A6NLU5|non_ion
>Q99550|non_ion
>Q9NYV7|non_ion

>Q61476|non_ion
>Q09712|non_ion
>Q8VXX9|non_ion
>G5EEE1|non_ion
>O75326|non_ion
>P0AC78|non_ion
>Q9Z309|non_ion
>P9WHR9|non_ion
>O54967|non_ion
>Q9UQ53|non_ion
>Q9CA75|non_ion
>Q6BZZ1|non_ion
>Q9XIC5|non_ion
>Q6C994|non_ion
>Q62147|non_ion
>O94537|non_ion
>Q96262|non_ion
>B9X187|non_ion
>Q54VX4|non_ion
>Q00246|non_ion
>P53320|non_ion
>H2KZM6|non_ion
>Q8GWA7|non_ion
>P86936|non_ion
>Q17388|non_ion
>P0CD99|non_ion
>P9WP15|non_ion
>O55227|non_ion
>P97207|non_ion
>O94305|non_ion
>Q8HZQ5|non_ion
>Q9V6G5|non_ion
>Q9SRQ7|non_ion
>P35991|non_ion
>P0AFN2|non_ion
>Q8WV93|non_ion
>Q3UFQ8|non_ion
>Q6ZQA6|non_ion
>H2QL32|non_ion
>Q9JI51|non_ion
>O31603|non_ion
>P0ADP5|non_ion
>O45797|non_ion
>Q94AU2|non_ion
>Q13574|non_ion
>Q757K7|non_ion
>Q7L8C5|non_ion
>Q08109|non_ion
>Q66T02|non_ion
>Q8BX90|non_ion
>Q7Z6P3|non_ion
>Q39085|non_ion
>Q9NZC2|non_ion
>Q641M3|non_ion
>Q8NBV8|non_ion

>Q6R2K3|non_ion

>Q80XG9|non_ion

>Q75D34|non_ion

>Q8N6G5|non_ion

>Q8I4N4|non_ion

>P0AD14|non_ion

>Q08722|non_ion

>Q04164|non_ion

>Q9SJG2|non_ion

>Q80X71|non_ion

>P56485|non_ion

>Q9R0Z9|non_ion

>Q8IDR3|non_ion

>Q8WW52|non_ion

>P14377|non_ion

>Q9XUS7|non_ion

>Q9JK66|non_ion

>O53176|non_ion

>P26234|non_ion

>P75830|non_ion

>Q9SFB0|non_ion
